# The Effect of Crystal Face of Fe_2_O_3_ on the Electrochemical Performance for Lithium-ion Batteries

**DOI:** 10.1038/srep29381

**Published:** 2016-07-06

**Authors:** Minmin Chen, Enyue Zhao, Qingbo Yan, Zhongbo Hu, Xiaoling Xiao, Dongfeng Chen

**Affiliations:** 1 ^1^College of Materials Science and Opto-electronic Technology University of Chinese Academy of Sciences, Beijing 100049, P. R. China; 2 China Institute of Atomic Energy, Beijing 102413, P. R. China

## Abstract

Fe_2_O_3_ nanorods exposing (001) and (010) plane as well as Fe_2_O_3_ nanosheets exposing (001) plane have been successfully synthesized. Fe_2_O_3_ nanosheets exhibit better cycle performance and rate capabilities than that of Fe_2_O_3_ nanorods. The discharge capacity of Fe_2_O_3_ nanosheets can stabilize at 865 mAh/g at the rate of 0.2 C (1C = 1000 mA/g) and 570 mAh/g at the rate of 1.2 C after 80 cycles, which increased by 90% and 79% compared with 456 mAh/g and 318 mAh/g of Fe_2_O_3_ nanorods. In comparison with (010) plane, the (001) plane of hematite possesses larger packing density of Fe^3+^ and O^2−^, which is responsible for the superior electrochemical performances of Fe_2_O_3_ nanosheets than that of Fe_2_O_3_ nanorods. In addition, potentiostatic intermittent titration (PITT) results show the diffusion coefficients of Li^+^ (D_Li_) of Fe_2_O_3_ nanosheets is higher than that of Fe_2_O_3_ nanorods. The higher diffusion coefficients of Li^+^ is favorable for the excellent lithium-storage capabilities and rate capability of Fe_2_O_3_ nanosheets. Inspired by our results, we can design and synthesize Fe_2_O_3_ or other electrodes with high performances according to their structure features in future.

3d transition-metal oxides, which can be used as anode materials, such as iron oxide, cobalt oxide, and nickel oxide have attracted a great deal of attentions for their much higher capacity than that of conventional graphite (372 mAhg^−1^)^1^,^2^,^3^,^4^,^5^,^6^,^7^,^8^,^9^,^10^,^11^,^12^. For instance, the theoretical capacity of Co_3_O_4_ is about 890 mAhg^−1^, which is almost two and a half times higher than that of graphite. However, high price and toxicity of Co limit the application of Co_3_O_4_^13^,^14^. Interestingly, Fe_2_O_3_ also exhibits high capacity (1007 mAhg^−1^) like Co_3_O_4_. More importantly, due to its low cost, nontoxicity and high resistance to corrosion, Fe_2_O_3_ has attracted special attentions in recent years^15^,^16^. For example, various morphologies of Fe_2_O_3_ such as nanoparticles, nanotubes, hollow structure and thin films have been studied as electrodes for lithium-ion batteries^17^,^18^,^19^,^20^,^21^,^22^,^23^,^24^,^25^. Lou *et al*. prepared a series of hollow microspheres of iron oxides which showed significantly improved lithium-storage capabilities^24^,^25^. In addition to the above-mentioned methods, many studies have proved that the crystal plane structure of electrode materials has a significant effect on the electrochemical properties. Islam *et al*. reported that the (010) plane of LiFePO_4_ is a favorable plane for fast Li^+^ transport^26^. Wei *et al*. found that the electrochemical performance of lithium rich material Li(Li_0.17_Ni_0.25_ Mn_0.58_)O_2_ with (010) and (100) planes have been greatly increased, exhibiting not only a high reversible capacity but also an excellent cycle stability^27^. Huang *et al*. found the facet-dependent electrochemical properties of Co_3_O_4_ toward heavy metal ions and found that the Co_3_O_4_ nanoplates with (111) facet performed better electrochemical sensing capability than the Co_3_O_4_ nanocubes with (001) facet^28^. Not long ago, we also reported the facet-dependent electrochemical capability of Co_3_O_4_ as anode material for Li-ion batteries and proved the Co_3_O_4_ octahedron with exposed (111) plane exhibited more excellent electrochemical properties than that of Co_3_O_4_ cube with exposed (001) plane and Co_3_O_4_ truncated octahedron with exposed (001) and (111) planes^29^. Therefore, studies on the crystal plane controllable synthesis of nanomaterials are of great interest and are actively being pursued. So, controlling the exposed crystal plane of Fe_2_O_3_ might also be an effective strategy to further improve the electrochemical performance of Fe_2_O_3_ as anode materials for lithium-ion batteries.

In this article, we successfully synthesized two kinds of Fe_2_O_3_ with exposed different crystal plane, including nanorods with (001) and (010) plane and nanosheets with the (001) plane. Interestingly, when used as anode materials in lithium-ion batteries, Fe_2_O_3_ nanosheets exhibit better cycle performance and rate capabilities than that of Fe_2_O_3_ nanorods. To be specific, the discharge capacity of Fe_2_O_3_ nanosheets could stabilize at 865 mAhg^−1^ at the rate of 0.2C (1 C = 1000 mAg^−1^) and 570 mAhg^−1^ at the rate of 1.2 C over 80 cycles, which increased by 90% and 79% compared with 456 mAhg^−1^ and 318 mAhg^−1^ of Fe_2_O_3_ nanorodes. Herein, the outstanding electrochemical performance of Fe_2_O_3_ nanosheets can be attributed to the highly exposed (001) planes. Crystal structure have revealed that the (001) plane possesses larger packing density than that of (010) plane, and the crystal effect is the crucial reason for the differences of electrochemical performance^30^. On the other hand, potentiostatic intermittent titration (PITT) results show that Fe_2_O_3_ nanosheets have higher diffusion coefficient of Li^+^ (D_Li_) and are more favorable for the diffusion of lithium ion.

To the best of our knowledge, we, for the first time, combined electrochemical experiment and crystal structure analysis to elucidate exposed crystal plane-electrochemical properties relationship of Fe_2_O_3_ as anode for rechargeable lithium ion batteries. Our results indicate the superior electrochemical performances of Fe_2_O_3_ nanosheets can be attributed to (1) the larger packing density of Fe^3+^ and O^2−^ of (010) plane and (2) the higher diffusion coefficient of Li^+^ (D_Li_) of Fe_2_O_3_ nanosheets during discharge-charge process. Furthermore, our results provide a idea which we can design and synthesize electrode materials with high performances according to their structure features in future.

## Results

**Figure 1:**
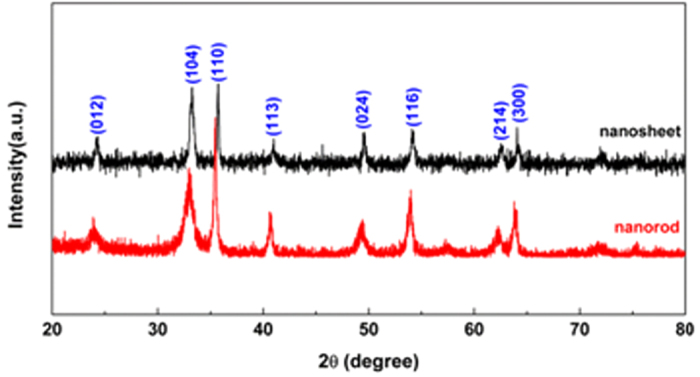
Powder X-ray diffraction patterns of Fe_2_O_3_ nanorods and nanosheets in a 2θ range of 10–80°.

**Figure 2:**
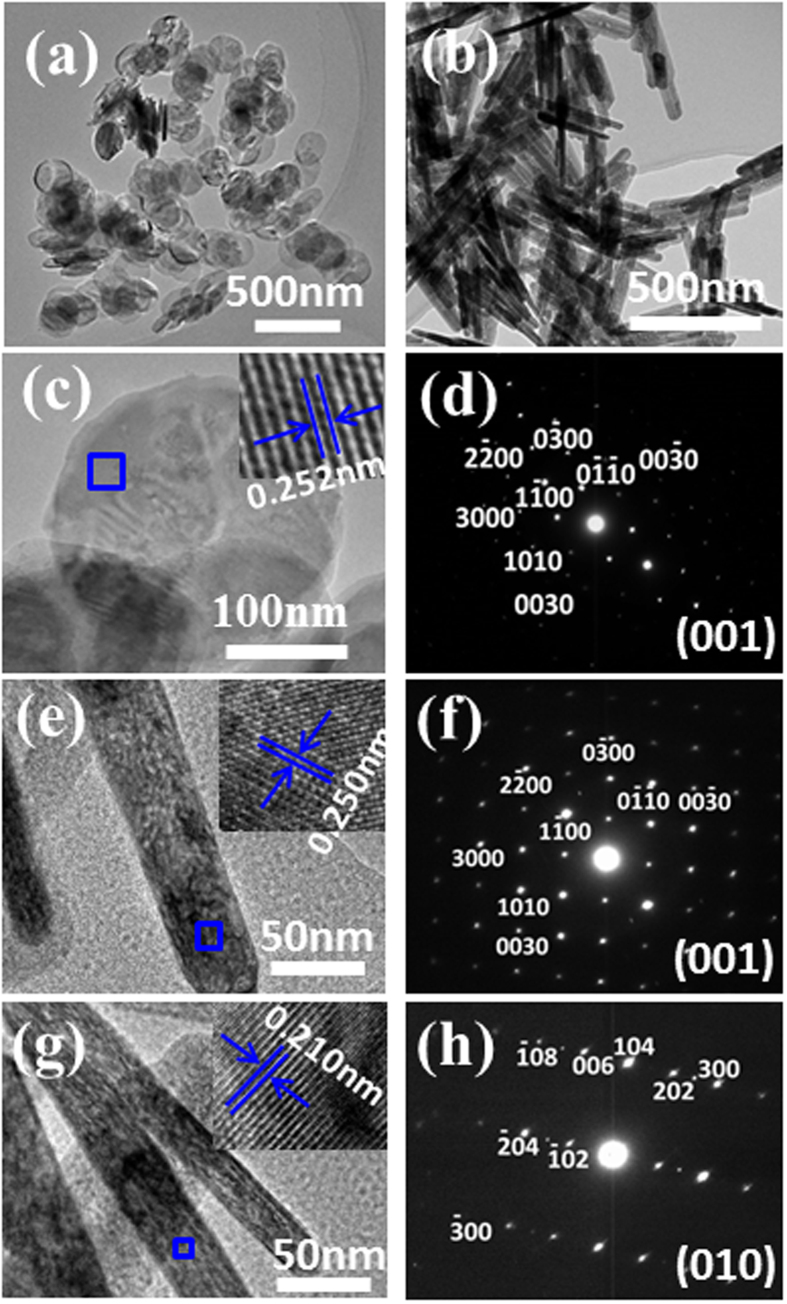
TEM images of Fe_2_O_3_ nanosheets (**a**) and Fe_2_O_3_ nanorods (**b**); (**c,d**) The TEM image of a Fe_2_O_3_ nanosheet, inset shows the lattice fringes and the corresponding SAED pattern; (**e,f**) The TEM image of Fe_2_O_3_ nanorode with {001} plane, inset shows the lattice fringes and the corresponding SAED pattern; (**g,h**) The TEM image of Fe_2_O_3_ nanorode with {010} plane, inset shows the lattice fringes and the corresponding SAED pattern.

In Fig. 1, the indexed X-Ray Diffraction (XRD) patterns of Fe_2_O_3_ samples show that the diffraction peaks match well with the standard PDF card (JCPDS no. 86–2368), indicating the purity of the products and the two kinds of Fe_2_O_3_ belong to the same space group. The exposed facets of nanosheets and nanorodes have been determined by high resolution transmission electron microscopy (HRTEM) characterization in Fig. 2c–h. The clear lattice spacing and fast Fourier transform selected-area electron diffraction (FFT-SAED) patterns indicate that Fe_2_O_3_ nanosheets and nanorods are single crystalline. Figure 2c shows the TEM image of a Fe_2_O_3_ nanosheet, and the corresponding SAED pattern is shown in Fig. 2d. It can be clearly seen that the exposed crystal facet is perpendicular to the (3000), (0300) and (0030) facets of Fe_2_O_3_ nanosheets, and the interlayer spacings of 0.252 nm inserted in Fig. 1c correspond to the (110) plane of the Fe_2_O_3_. Thus it can be concluded that the exposed facets of the nanosheets are (001). Figure 2e and f show the similar interlayer spacings and SAED pattern compared with the Fe_2_O_3_ nanosheets, which indicate that one of the exposed facets of the Fe_2_O_3_ nanorods are (001). Figure 2g shows the TEM image of another facet of Fe_2_O_3_ nanorodes and the corresponding SAED pattern is shown in Fig. 2h. The SAED pattern in Fig. 2h shows that the exposed crystal facet is perpendicular to the (300), (006) and (202) facets of the Fe_2_O_3_, and the interlayer spacings of 0.210 nm inserted in Fig. 2g correspond to the (202) plane of the Fe_2_O_3_.

**Figure 3:**
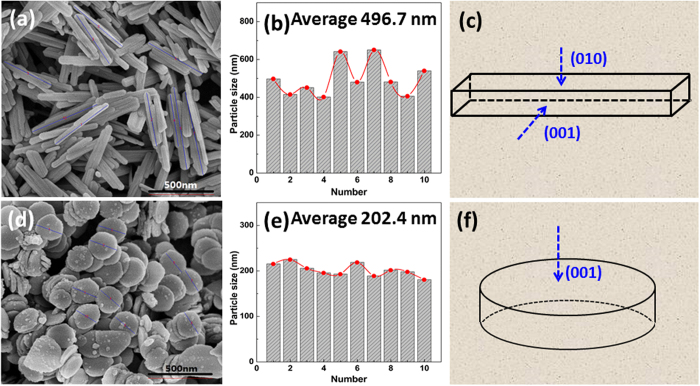
The SEM images and crystal size distribution histogram of (**a,b**) Fe_2_O_3_ nanorods and (**d,e**) Fe_2_O_3_ nanosheets; Structural models of (**c**) Fe_2_O_3_ nanorods and (**f**) Fe_2_O_3_ nanosheets.

So another exposed facets of the nanorodes are (010). The structural models of Fe_2_O_3_ nanorod is displayed in Fig. 3c, the exposed (001) and (010) crystal facets can be clearly shown and the models of Fe_2_O_3_ nanosheet is showed in Fig. 3f. Figure 3a,d show the SEM images of Fe_2_O_3_ nanorodes and nanosheets, respectively. It can be seen that the average length of Fe_2_O_3_ nanorods is about 500 nm, the width and thickness is about 50 and 15 nm, respectively. The average diameter and thickness of Fe_2_O_3_ nanosheets is about 200 nm and 15 nm, respectively. The Fe_2_O_3_ nanosheets and nanorods of nanosize can reduce the diffusion length of Li^+^ ions and increase reactivity of the material, which are very favorable for excellent electrochemical performances.

**Figure 4:**
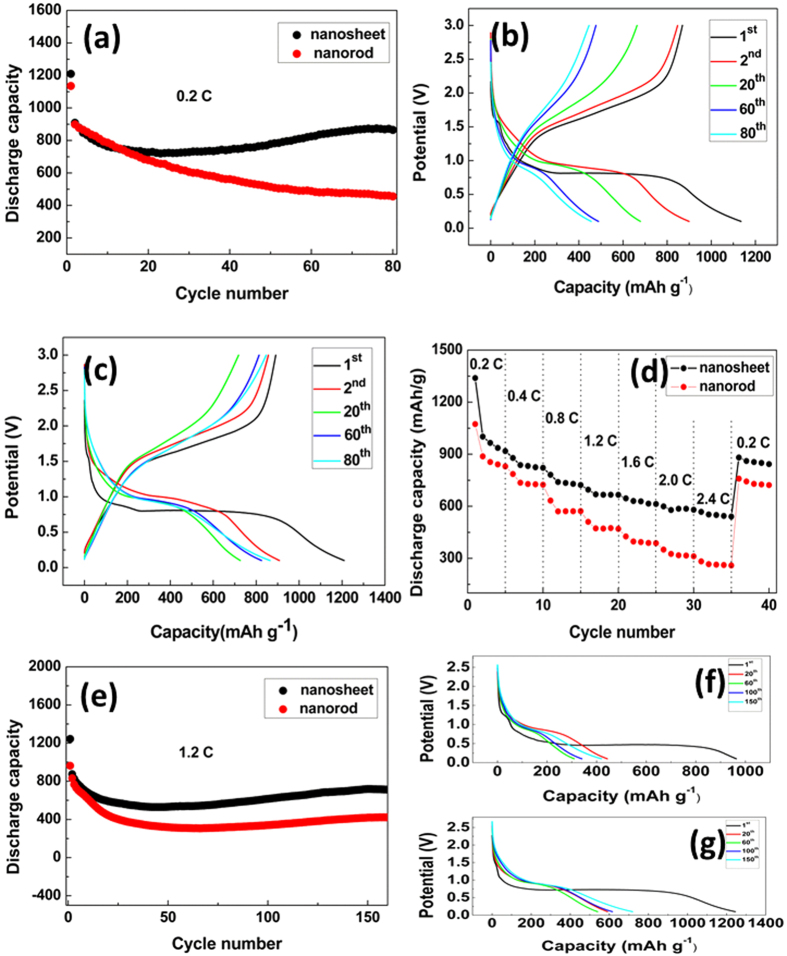
N_2_ absorption curves of Fe_2_O_3_ nanosheets and Fe_2_O_3_ nanorods. (**a**) Comparison of cycling performance of the two kinds of Fe_2_O_3_ at the rate of 0.2 C; The charge/discharge curves of (**b**) Fe_2_O_3_ nanorods and (**c**) Fe_2_O_3_ nanosheets in the 1st, 2nd, 20th, 60th and 80th cycles at the rate of 0.2 C, respectively. (**d**) The rate performances of Fe_2_O_3_ nanorods and Fe_2_O_3_ nanosheets. (**e**) Plots of the specific discharge capacity vs. cycle number for the Fe_2_O_3_ nanostructure electrode at the rate of 1.2 C; The discharge curves of (**f**) Fe_2_O_3_ nanorods and (**g**) Fe_2_O_3_ nanosheets in the 1st, 20th, 60th, 100th and 150th cycles at the rate of 1.2 C, respectively.

Subsequently, the comparison galvanostatic discharge capacities of Fe_2_O_3_ nanorods and nanosheets in a potential range of 0.1–3.0 V (vs Li/Li^+^) at a rate of 0.2 C were comprehensively investigated and illustrated in Fig. 4a–c. It can be seen from Fig. 4a that Fe_2_O_3_ nanorod and Fe_2_O_3_ nanosheet electrodes deliverer approximately a discharge capacity of 1135 mAhg^−1^ and 1210 mAhg^−1^ in the first cycle, respectively. After that the discharge capacity decreases rapidly, and the reason can be ascribed to the change of structure during the initial charge-discharge process. From the beginning of the second cycle, two kinds of Fe_2_O_3_ electrodes exhibit good cycle stability until to the 20 cycles. Surprisingly, after 20 cycles, the discharge capacity of the Fe_2_O_3_ nanorods monotonically decline, while the discharge capacity of Fe_2_O_3_ nanosheets slightly increase. Similar phenomenon also has been found in the case of CoO and Co_3_O_4_ as well as other Fe_2_O_3_ reports in the literature, though a clear understanding has not been obtained^31^,^32^,^33^. As shown in Fig. 4a, the discharge capacity of the Fe_2_O_3_ nanosheets maintains at 865 mAhg^−1^ with a capacity retention of 95.3% after 80 cycles, in contrast, the discharge capacity of Fe_2_O_3_ nanorods maintains at 456 mAh/g with a capacity retention of 50.7% after 80 cycles.

Figure 4d shows the rate performance of Fe_2_O_3_ nanorods and nanosheets. Specifically, the discharge capacity of Fe_2_O_3_ nanorods at 0.2, 0.4, 0.8, 1.2, 1.6, 2.0 and 2.4 C are 896, 763, 627, 544, 478, 430 and 385 mAhg^−1^, respectively. The corresponding values for the Fe_2_O_3_ nanosheets were 966, 832, 734, 667, 628, 586 and 550 mAhg^−1^, respectively. By comparing the discharge capacity of the two samples, Fe_2_O_3_ nanosheets display higher capacity than Fe_2_O_3_ nanorods at various charge−discharge rates from 0.2 to 2.4 C. Meanwhile, as the growth of charge–discharge current density, the gap between the discharge capacities of the Fe_2_O_3_ nanorods and nanosheets samples became larger. For instance, the discharge capacity of Fe_2_O_3_ nanosheets increase by 8% compared with that of Fe_2_O_3_ nanorods at 0.2 C, while the discharge capacity increase by 43% at the rate of 2.4 C. In addition, it should be noted when the rate was returned back to the 0.2 C, Fe_2_O_3_ nanosheets still show higher discharge capacity than that of Fe_2_O_3_ nanorods. At the recovery rate of 0.2 C, both Fe_2_O_3_ nanosheets and nanorods display lower discharge capacity compared with the initial capacity at 0.2 C. The phenomenon is due to the destruction of crystal structure of Fe_2_O_3_ during discharge-charge cycle process.

In order to research the cycle stability under high current density, the Fe_2_O_3_ nanorods and nanosheets are tested at the rate of 1.2 C, as shown in Fig. 4e–g. Obviously, the electrode of Fe_2_O_3_ nanosheets shows much higher discharge capacity than that of Fe_2_O_3_ nanorods at high rate. Especially, the discharge capacity of Fe_2_O_3_ nanosheets can reach 719 mAhg^−1^ after 150 cycles. This value is 71% higher than that of Fe_2_O_3_ nanorods, which only shows 419 mAhg^−1^ after 150 cycles.

**Figure 5:**
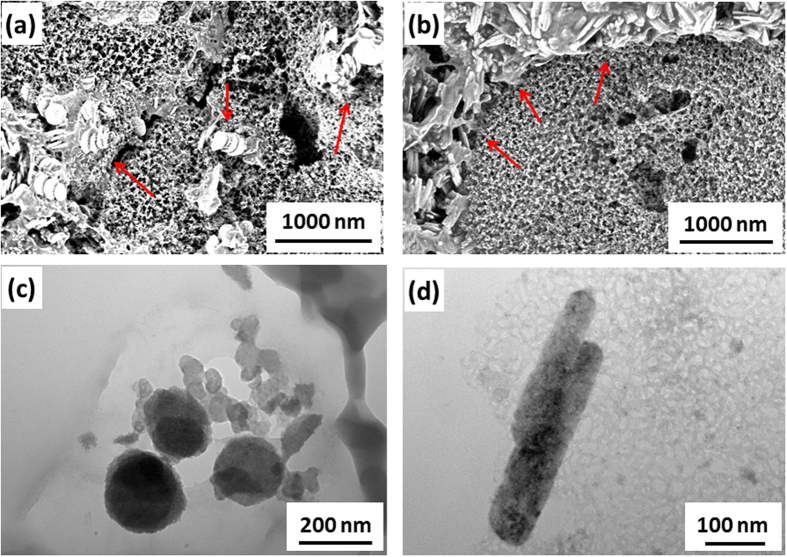
SEM and TEM images of (**a,c**) Fe_2_O_3_ nanosheets and (**b,d**) Fe_2_O_3_ nanorods after 30 cycles at 0.2 C.

SEM and TEM images of Fe_2_O_3_ nanosheets and nanorods samples after extensive cycling are shown in Fig. 5. It can be clearly seen in Fig. 5a,c that Fe_2_O_3_ nanosheets keep relatively complete sheet structure after extensive charge-discharge cycling. Similar to nanosheets, Fe_2_O_3_ nanorods also show well virgulate shape which can be seen in Fig. 5b,d. In addition, there is no significant change of the Fe_2_O_3_ particle size after charge-discharge cycling.

**Figure 6:**
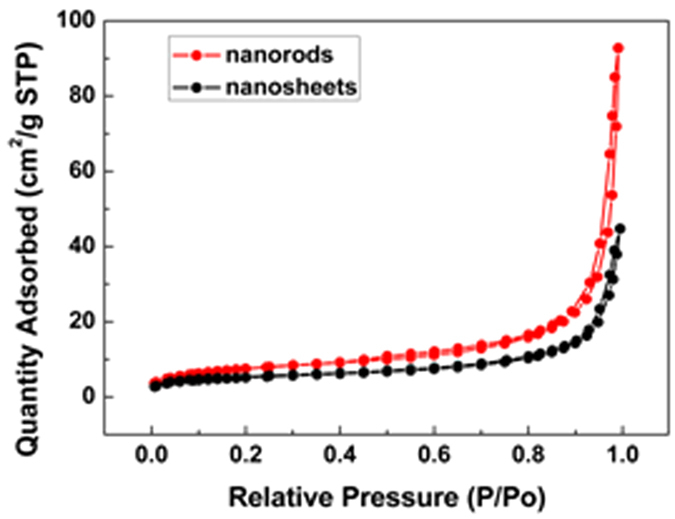
N2 absorption curves of Fe_2_O_3_ nanosheets and Fe_2_O_3_ nanorods.

It is reported that the Brunauer–Emmett–Teller (BET) surface areas of electrode materials play a improtant role on the electrochemical performance of lithium ions batteries^34^. Our nitrogen-sorption analysis reveals that the BET specific surface areas of Fe_2_O_3_ nanorods and Fe_2_O_3_ nanosheets were 26.81 and 18.25 m^2^/g, respectively (Fig. 6). The BET specific surface areas of Fe_2_O_3_ nanorods is larger than that of Fe_2_O_3_ nanosheets, whereas, the Fe_2_O_3_ nanosheets exhibit better electrochemical properties compared with Fe_2_O_3_ nanorods. So it can be concluded that the effect of specific surface areas of electrodes on the difference of electrochemical properties between Fe_2_O_3_ nanosheets and nanorodes can be overlooked.

**Figure 7:**
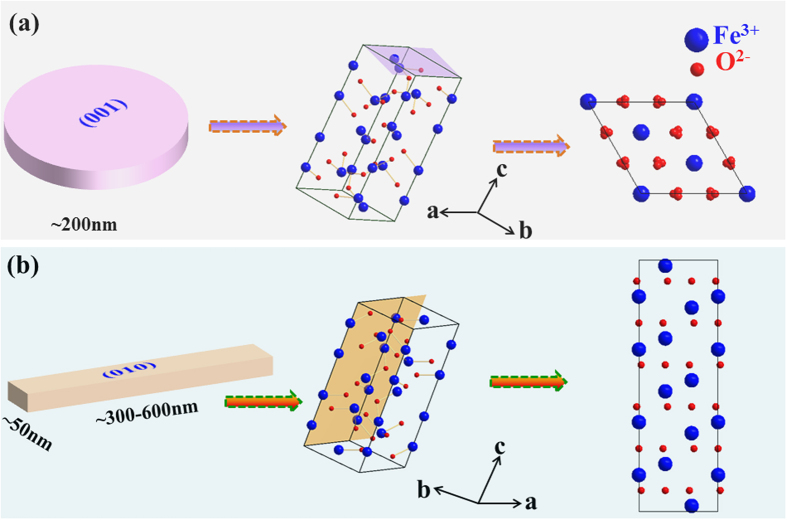
The surface atomic configurations in (**a**) the (001) plane and schematic hematite structure projected along {001}, (**b**) the (010) plane and schematic hematite structure projected along {010}. The Fe_2_O_3_ Crystallographic Information File (CIF) was taken from the NIST/FIZ FindIt Inorganic Crystal Structure Database.

Evidently, the electrochemical performances of lithium ion batteries are related to the intrinsic crystal structure^35^. So the crystal structure of Fe_2_O_3_ is analyzed. For Fe_2_O_3_ samples, the (001) plane has been found possessing the larger packing density, in which Fe^3+^ and O^2−^ ions pack layer by layer. Specifically, the packing densities of the Fe^3+^ and O^2−^ are 9.11 nm^−2^ and 13.8 nm^−2^, respectively. In contrast, the packing densities of the (010) facets for ions are 2.89 nm^−2^ and 5.78 nm^−2^. Due to the high atomic density, more Fe^3+^ ions participate in the reaction, and lead to a high specific capacity^28^. The detailed crystal structure of Fe_2_O_3_ have been displayed in Fig. 7. Meanwhile, it can be seen from the model of Fe_2_O_3_ nanosheets and nanorodes in Fig. 7, that the proportion of (001) plane in nanosheet is almost 100%, while in nanorodes is about 23%. And the mainly exposed crystal plane is (010) facet in nanorodes, in which the proportion of (010) plane is about 77%. The results indicate that the Fe_2_O_3_ samples which exposed more (001) plane show a superior electrochemical capability.

**Figure 8:**
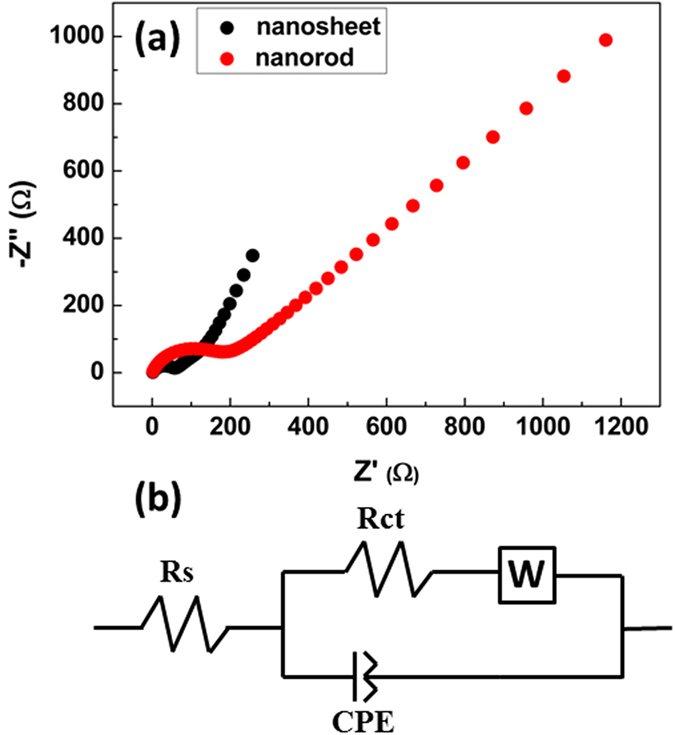
(**a**) Nyquist plots for Fe_2_O_3_ nanorods and Fe_2_O_3_ nanosheets; (**b**) The corresponding equivalent circuit.

Figure 8 shows Nyquist plots of the two kinds of Fe_2_O_3_ electrode measured at the open circuit potential and an equivalent circuit proposed to fit the spectra. As can be seen from Table 1, the charge transfer resistances (R_ct_) for Fe_2_O_3_ nanosheets (53 Ω) is much smaller than that obtained from the Fe_2_O_3_ nanorodes (179 Ω) electrode. The electrochemical impedance spectroscopy (EIS) data indicates that Fe_2_O_3_ nanosheets possesses smaller lithium ion migration resistance and is more conducive to the rapid migration of lithium ions.

**Figure 9:**
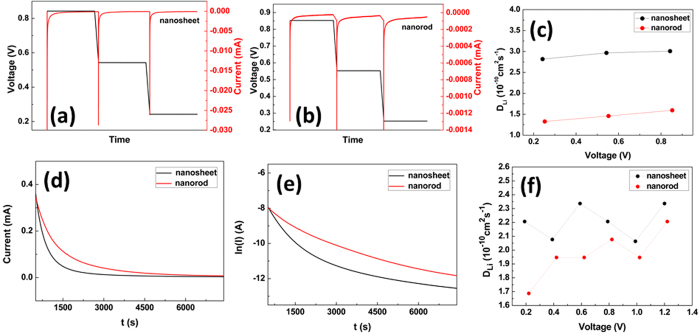
PITT curves of (**a**) Fe_2_O_3_ nanosheets and (**b**) Fe_2_O_3_ nanorods and (**c**) diffusion coefficients of Li^+^ (D_Li_) before discharge-charge cycle; Current-time transient plots of PITT for a potential step of 1.0–0.8 V after one cycle, (**d**) I vs. t and (**e**) ln(I) vs. t; (**f**) D_Li_ of Fe_2_O_3_ nanorods and Fe_2_O_3_ nanosheets during discharge processe.

For the sake of confirming D_Li_ in electrode materials, PITT measurement was performed. Figure 9a–c display the PITT results of Fe_2_O_3_ samples before discharge-charge cycle. It can be seen the D_Li_ of Fe_2_O_3_ nanosheets is about one time higher than that of Fe_2_O_3_ nanorods. Figure 9d–f display the PITT results of Fe_2_O_3_ samples after a circle of discharge-charge cycle at the current density of 200 mA/g. It is obviously that D_Li_ of Fe_2_O_3_ nanosheets are higher than that of Fe_2_O_3_ nanorods. For instance, the D_Li_ average value of Fe_2_O_3_ nanosheets is 2.2 × 10^−10^ cm^2^ s^−1^ which increased by 15.7%, compared to 1.9 × 10^−10^ cm^2^ s^−1^ of Fe_2_O_3_ nanorods. The improved kinetic parameters D_Li_, indicate that Fe_2_O_3_ nanosheets possess higher lithium diffusion coefficient. For this reason, Fe_2_O_3_ nanosheets show better rate capability, as shown in Fig. 4d. Figure 4d show that Fe_2_O_3_ nanosheets with (001) planes possess higher discharge capacity not only at the low rate of 0.2 C but also at the high rate of 1.2 C. Additional, the Fe_2_O_3_ nanosheets with (001) planes exhibit better cycle stability and rate ability. Generally, the Fe_2_O_3_ nanosheets with (001) plane exhibit better electrochemical properties than that of the Fe_2_O_3_ nanorods with (010) and (001) planes.

In conclusion, we successfully synthesized two kinds of morphology of single crystal Fe_2_O_3_ with exposed different crystal plane, including nanorods with (001) and (010) plane and nanosheets with the (001) plane. Fe_2_O_3_ nanosheets exhibit better cycle performance and rate capabilities than that of Fe_2_O_3_ nanorods. The reasons can be attributed to that (1) the larger packing density of Fe^3+^ and O^2−^ of (010) plane and (2) the higher diffusion coefficient of Li^+^ (D_Li_) of Fe_2_O_3_ nanosheets during discharge-charge process. Our studies indicate that the crystal structure has a very important influence on the electrochemical performances, which may be helpful for developing high performance lithium ion batteries.

## Methods

### Materials synthesis

The Fe_2_O_3_ nanorods were synthesized by FeOOH nanorods template. To prepare FeOOH nanorods precursors, 1.9 mmol of FeCl_3_·6H_2_O was put into 15 ml of deionized water to form a homogeneous solution, then added 15 ml of 0.8 M NaOH solution under stirring, quickly. After stirring for 10 min, the total solution was transferred into a 50 ml Teflon-lined stainless steel autoclave, sealed and heated at 180 °C for 4 h. The result product was collected by centrifugation, washed with deionized water and ethanol, then was dried at 80 °C and calcined at 250 °C for 2 h. The Fe_2_O_3_ nanosheets were synthesized based on the previous work^28^. 5.1 mmol of FeCl_3_·6H_2_O was dissolved in 35 ml of anhydrous ethanol, and 38.4 mmol of CH_3_COONa·3H_2_O was rapidly added into the solution with stirring. After about 5 min of stirring, all of the reactants were transferred into a 50 ml Teflon-lined stainless steel autoclave, sealed and heated at 200 °C for 22 h. The result product was collected by centrifuge, washed with deionized water and ethanol. Then dried at 80 °C and calcined at 250 °C for 2 h.

### Characterization

XRD measurements were performed on a Persee XD2 X-ray diffractometer with Cu-Kα radiation (λ = 1.5418). The size and morphology of all of the samples were measured with a S-4800 HITACHI scanning electron microscope (SEM) and a JEM-2100 transmission electron microscope (TEM). The specific surface areas of the powders were collected by a Gemini V Brunauer-Emmett-Teller (BET).

### Electrochemical Measurement

For electrochemical studies, working electrode was fabricated with mixing active material, acetylene black and polyvinylidene fluoride (PVDF) with weight ratio 2:1:1 using N-methylpyrrolidone (NMP) as solvent. The slurry was fully ground and pasted onto copper foil, and then the loaded copper foil was dried in a vacuum oven at 120 °C for 12 h. Lithium metal, celgard 2300 membrane and 1 M LiPF_6_ solution in DMC/EC (1: 1 in volume) were used as counter electrode, separator and electrolyte respectively to assemble coin cells in an Ar-filled glove box. The galvanostatic charge/discharge performance of the cells were tested on a battery testing system (BTS-5 V 5 mA, Neware) with the voltage between 0.1 and 3.0 V at the current density of 200, 400, 800, 1200, 1600, 2000, 2400 mA/g. The electrochemical spectroscopy (EIS) was tested with an (PGSTAT302N, Metrohm-Autolab) instrument using an amplitude of 5 mV and a frequency range from 100 KHz to 0.1 Hz. The PITT tests were also performed on the same instrument with EIS.

## Additional Information

**How to cite this article**: Chen, M. *et al*. The Effect of Crystal Face of Fe_2_O_3_ on the Electrochemical Performance for Lithium-ion Batteries. *Sci. Rep.* **6**, 29381; doi: 10.1038/srep29381 (2016).

## Figures and Tables

**Table 1:** The fitted solution resistances (R_s_) and charge transfer resistances (R_ct_) for the Nyquist plots of Fe_2_O_3_ nanorods and nanosheets.

Sample	R_S_		R_ct_	
	Value (Ω)	Error %	Value (Ω)	Error %
Fe_2_O_3_ nanorods	1.2388	26.4	178.7	4.346
Fe_2_O_3_ nanosheets	1.7051	6.436	53.337	2.482
